# Strawberry-Tree Honey Induces Growth Inhibition of Human Colon Cancer Cells and Increases ROS Generation: A Comparison with Manuka Honey

**DOI:** 10.3390/ijms18030613

**Published:** 2017-03-11

**Authors:** Sadia Afrin, Tamara Y. Forbes-Hernandez, Massimiliano Gasparrini, Stefano Bompadre, José L. Quiles, Gavino Sanna, Nadia Spano, Francesca Giampieri, Maurizio Battino

**Affiliations:** 1 Department of Clinical Sciences, Faculty of Medicine, Polytechnic University of Marche, via Ranieri 65, Ancona 60131, Italy; dolla.bihs@gmail.com (S.A.); tamara.forbe@gmail.com (T.Y.F.-H.); m.gasparrini@univpm.it (M.G.); 2 Department of Biomedical Sciences and Public Health, Faculty of Medicine, Polytechnic University of Marche, via Ranieri 65, Ancona 60131, Italy; s.bompadre@univpm.it; 3 Department of Physiology, Institute of Nutrition and Food Technology “José Mataix”, Biomedical Research Centre, University of Granada, Armilla 18100, Spain; jlquiles@ugr.es; 4 Department of Chemistry and Pharmacy, University of Sassari, via Vienna 2, Sassari 07100, Italy; sanna@uniss.it (G.S.); nspano@uniss.it (N.S.); 5 Centre for Nutrition & Health, European University of the Atlantic, Santander 39011, Spain; 6 Department of Clinical Sciences, Faculty of Medicine, Polytechnic University of Marche, via Ranieri 65, Ancona 60131, Italy

**Keywords:** Manuka honey, strawberry tree honey, polyphenols, antioxidant activity, cytotoxicity, reactive oxygen species, colon cancer

## Abstract

Honey is a natural product known to modulate several biological activities including cancer. The aim of the present study was to examine the phytochemical content and the antioxidant activity of Strawberry tree (*Arbutus unedo*) honey (STH) and its cytotoxic properties against human colon adenocarcinoma (HCT-116) and metastatic (LoVo) cell lines in comparison with Manuka (*Leptospermum scoparium*) honey (MH). Several unifloral STH and MH were analyzed for their phenolic, flavonoid, amino acid and protein contents, as well as their radical scavenging activities. STH from the Berchidda area showed the highest amount of phenolic, flavonoid, amino acid and protein content, and antioxidant capacity compared to MH. Both STH and MH induced cytotoxicity and cell death in a dose- and time-dependent manner in HCT-116 and LoVo cells, with less toxicity on non-cancer cells. Compared to MH, STH showed more effect at lower concentrations on HCT-116 and LoVo cells. In addition, both honeys increased intracellular reactive oxygen species (ROS) generation. In HCT-116 cells, STH and MH induced similar ROS production but in LoVo cells STH induced a higher percentage of ROS compared to MH. Our results indicate that STH and MH can induce cell growth inhibition and ROS generation in colon adenocarcinoma and metastatic cells, which could be due to the presence of phytochemicals with antioxidant properties. These preliminary results are interesting and suggest a potential chemopreventive action which could be useful for further studies in order to develop chemopreventive agents for colon cancer.

## 1. Introduction

In Europe, colorectal cancer is the second most common cancer [1] while over one million new cases are detected each year worldwide [2]. The survival rate of colon cancer is only five years due to its resistance to cytostatic drugs [3]. However, no proper treatment options are available for this type of cancer. Therefore, there is an urgent need to establish novel preventive and therapeutic approaches for this disease. Natural compounds have the potential to treat for colorectal cancer by inducing the cytotoxic effect on colon cancer cells while they are less toxic to normal colonic epithelial cells [4,5]. Honey is a good source of biological or pharmacological compounds with antioxidant, antibacterial, anti-inflammatory, antihypertensive, hypoglycemic, anti-proliferative, anticancer and anti-metastatic activities [6,7,8,9,10,11,12]. In this case, it has to be taken into consideration that the bioactive compounds of honey are rather variable, and depend not only on the honey’s floral sources, but also on the geographical origins, as well as on seasonal and environmental factors which may be accountable for the detected variations.

Strawberry tree (*Arbutus unedo* L., Family: Ericaceae) unifloral honey is a typical and renowned product of certain Mediterranean regions, such as Sardinia. However, despite its high reputation, there are insufficient data on its phytochemical composition or biological properties. Only a few studies have investigated the organic acid profile of strawberry tree honey (STH), and its melissopalynological and physicochemical properties [13,14,15,16,17]. STH expresses exceptional antioxidant properties due to its high amounts of phenolic compounds, mainly flavonoids and phenolic acids [18,19]. Homogentisic acid (2,5-dihydroxyphenylacetic acid) is the main phenolic marker of the STH [20,21] and particularly known for its attractive antioxidant, antiradical and protective effects, such as defensive actions against thermal cholesterol degradation [13,22].

Manuka honey (MH) from New Zealand derived from the *Leptospermum scoparium* tree (Family: Myrtaceae) has been extensively studied for antibacterial and antioxidant activity, as well as for wound healing mechanisms due to a large quantity of physicochemical properties and attractive therapeutic molecules [8,23,24,25,26]. MH contains numerous phenolic compounds, including flavonoids (pinobanksin, pinocembrin, chrysin, luteolin, quercetin, 8-methoxykaempferol, isorhamnetin, kaempferol and galangin) [27], phenolic acids (phenylacetic acid, phenyllacticacid, 4-hydroxybenzoic acid, kojic acid, 2-methoxybenzoic acid, syringic acid, and 4-methoxyphenyllactic acid) and other compounds (methylsyringate, leptosin, glyoxal, 3-deoxyglucosulose and methylglyoxal) [8]. Several studies have reported that methylglyoxal induces non-peroxide antibacterial activity even at very low concentrations [23,28]. In a recent study, it was found that MH promotes a wound healing mechanism due to improvement of the antioxidant response by modulating the AMPK/Nrf2 signaling pathway and increases the activity of antioxidant enzymes superoxide dismutase and catalase [25]. In addition, it induces an anti-proliferative effect in colon cancer cells by modulating the apoptotic pathway [29].

Only a few studies have evaluated the anticancer activity of honey on human colon cancer in vitro by targeting the different molecular mechanisms [29,30,31,32,33,34]. Among these, only one study reported the cytotoxic effect of MH on colon cancer and, to date, there are no data on the biological effects of STH on cancer. The present study was designed to investigate the phytochemical composition and antioxidant content of STHs from different Sardinian origins and to compare these characteristics with MH values. In addition, we compared the cytotoxic effect and reactive oxygen species (ROS) modulation by both honeys on human colon carcinoma (HCT-116) and Dukes’ type C, grade IV, colon metastasis (LoVo) cell lines. In addition, we also observed the cytotoxic effects of both honeys on non-cancer cells (human dermal fibroblast (HDF)).

## 2. Results and Discussion

### 2.1. Phytochemical Content of STH and MH

To evaluate the phytochemical composition of STH and MH, total polyphenol content (TPC) and total flavonoid content (TFC) were determined. As shown in Table 1, significant differences (*p* < 0.05) among the different groups were observed for TPC (Berchidda > MH > Monti > Luras > Sadali > Olbia) and TFC (Berchidda > Monti > MH > Luras > Olbia > Sadali). STH from Berchidda area showed the highest content of TPC (1.00 ± 0.02 g GAE/kg) followed by the area of Monti (0.86 ± 0.01 g GAE/kg), Luras (0.77 ± 0.02 g GAE/kg), and Sadali (0.76 ± 0.02 g GAE/kg), while the lowest value corresponded to Olbia (0.69 ± 0.01 g GAE/kg). Compared to MH (0.89 ± 0.01 g GAE/kg), STH from Berchidda area showed the highest value, Monti area showed a similar value and the STHs from Luras, Sadali and Olbia areas had lower values. The values obtained in our study were very close to those obtained by Rosa et al. for STH [13] and Alzahrani et al. for MH [35]. TPC of STH from Berchidda area was also higher compared to previously reported Cuban honey such as amber honey [11], Malaysian honey such as tualang honey [36], Portuguese honey [37] and Algerian honey [38]. These results suggest that STH from Berchidda could have a high antioxidant potential.

In the case of TFC, STH from Berchidda (108.20 ± 2.69 mg CAE/kg) and Monti (92.86 ± 14.17 mg CAE/kg) areas showed higher values compared to the STHs from the areas of Luras (69.96 ± 3.62 mg CAE/kg), Sadali (65.74 ± 2.50 mg CAE/kg) and Olbia (66.18 ± 0.61 mg CAE/kg) (Table 1). The values obtained from Berchidda and Monti areas were quite similar to the values reported by Aazza et al. [39]. In addition, TFC of MH (71.90 ± 0.03 mg CAE/kg) was lower than the STH from Monti and Berchidda areas but the values were very close to those from the Luras, Sadali and Olbia areas (Table 1). The TFC reported by Alvarez-Suarez et al. was also similar to the values obtained in our study for MH [25]. However, TFC of STHs were also higher than the Linen vine honey [11], Algerian honey [38], Gelam honey and Tualang honey [40], and lower than the values reported for Portuguese honey [37] and sourwood honey [41]. STH from Berchidda area may show potential antioxidant capacity due to its elevated flavonoid concentrations.

### 2.2. Total Protein and Free Amino Acid Content of STH and MH

Honey protein content relies on the type of plant species: since it is variable, the protein content of honey can be characterized for the presence of enzymes introduced by the bees themselves, and others derived from the nectar [42]. The amino acid of honey could play an important role in its antioxidant activity [43]. Total protein and free amino acid content were determined by colorimetric methods, and results are shown in Table 2. Total protein content decreased in the order: Berchidda > MH > Monti, Olbia > Luras = Sadali. Free amino acid content decreased in the order: Berchidda > Monti > MH > Olbia > Luras > Sadali. STH from Berchidda area presented the highest concentration of total protein (0.07 ± 0.00 g BSA/100 g) and free amino acid content (51.67 ± 9.64 mg LE/100 g) than other areas (0.03 to 0.04 g BSA/100 g and 10.28 to 14.56 mg LE/100 g, respectively), and was also higher than MH contents (0.05 ± 0.00 g BSA/100 g and 14.34 ± 0.13 mg LE/100 g, respectively) (Table 2). These results correspond with the values obtained by Spano et al. who reported that free amino acid of STH ranged between 7.3 to 53.8 mg/100 g [17]. Moreover, the values of free amino acid and total protein obtained from MH were lower than the values reported by Moniruzzaman et al. [41].

Regarding the values obtained for the other STHs, only the STH from Monti area presented similar values of free amino acids compared to MH; all the others presented lower contents (Table 2). Moreover, the protein content of STH from Berchidda area was lower than the values reported for Algerian honey [38], Sourwood honey [41], Bangladeshi honeys [44] but higher than Linen vine honey and Christmas vine honey [11].

### 2.3. Total Antioxidant Capacity of STH and MH

Total antioxidant capacity (TAC) of STH and MH was quantified by ferric reducing antioxidant power (FRAP), TEAC (Trolox equivalent antioxidant capacity) and DPPH (Diphenyl-1-picrylhydrazyl) assays (Table 3). The FRAP, TEAC and DPPH content were found in the orders: Berchidda > Monti > Luras > Sadali > Olbia > MH; Berchidda > MH > Luras = Sadali = Olbia > Monti; and Berchidda > Monti, Luras, Sadali, Olbia > MH, respectively. TAC of STH from Berchidda area was 0.92 ± 0.02 mmol Fe(II)/100 g and 0.54 ± 0.00 mmol TE/100 g (FRAP), 0.39 ± 0.01 mmol TE/100 g (TEAC), and 0.20 ± 0.01 mmol TE/100 g (DPPH) (Table 3). The values were significantly higher (*p* < 0.05) than those obtained for samples from other areas (Monti, Luras, Sadali and Olbia) but were lower than values previously reported by Tuberoso et al. [18]. On the other hand, TAC of MH (0.29 ± 0.00 mmol Fe(II)/100 g and 0.14 ± 0.00 mmol TE/100 g (FRAP), 0.22 ± 0.00 mmol TE/100 g (TEAC), and 0.06 ± 0.00 mmol TE/100 g (DPPH)) was slightly lower than the values reported by other studies [41,45]. There were significant differences (*p* < 0.05) between FRAP values of the STHs and MH (Table 3), suggesting that they may have diverse antioxidant potentials. Similarly, STH from Berchidda area had the highest TEAC and DPPH values among all the investigated honeys, which specifies its significant antioxidant activity. It is noted that MH presented higher TEAC values and lower DPPH values than other Sardinian STHs (Table 3). Overall, the TAC of STH from Berchidda area was higher than the previously reported Cuban honey [11], Portuguese honey [37], Malaysian honey [40,41], and Algerian honey [38].

### 2.4. Correlations between Biochemical Parameters and Antioxidant Potentials of STH and MH

A significant correlation (*p* < 0.05) was found between biochemical and antioxidant parameters of the honeys (Table 4). There was a high correlation between the TPC and TFC (*r* = 0.856, *p* ≤ 0.03) (Table 4). Likewise, a similar correlation was found between TPC and TFC values (*r* = 0.831, *p* ≤ 0.05) in Cuban honey [11]. In Algerian honey, Khalil et al. also found a correlation between the TPC and TFC (*r* = 0.776, *p* ≤ 0.01) [38]. In our study, a strong correlation was also observed between FRAP and DPPH (*r* = 0.808, *p* ≤ 0.002), as well as TEAC and DPPH (*r* = 0.704, *p* ≤ 0.01). Simultaneously, a significant correlation was found between TPC and TEAC (*r* = 0.870, *p* ≤ 0.002), while low correlation coefficients were obtained between TPC and FRAP (*r* = 0.663, *p* ≤ 0.05) and DPPH (*r* = 0.678, *p* ≤ 0.05). In addition, high correlations were found between TFC and FRAP (*r* = 0.878, *p* ≤ 0.002), and TFC and DPPH (*r* = 0.796, *p* ≤ 0.009), while low correlations were obtained between TFC and TEAC (*r* = 0.678, *p* ≤ 0.04) (Table 4). In a previous study on Cuban honey, a significant correlation was found between TPC and TAC (*r* = 0.89, *p* ≤ 0.006 by FRAP and *r* = 0.96, *p* ≤ 0.001 by TEAC) and also between TFC and TAC (*r* = 0.89, *p* ≤ 0.05 by FRAP and *r* = 0.8315, *p* ≤ 0.05 by TEAC) [11]. Similarly, a positive correlation was observed on Malaysian honey between TPC and TAC (*r* = 0.761, *p* ≤ 0.01 by FRAP, *r* = 0.837, *p* ≤ 0.05 by TEAC and *r* = 0.789, *p* ≤ 0.05 by DPPH), and also TFC and TAC (*r* = 0.782, *p* ≤ 0.05 by FRAP, *r* = 0.735, *p* ≤ 0.05 by TEAC and *r* = 0.607, *p* ≤ 0.05 by DPPH) [41]. According to the correlation values, the results confirmed that the polyphenols and flavonoids significantly contribute to the TAC of honeys.

Moreover, scanty amounts of protein and amino acid are present in honey and they are significantly correlated between the TPC (*r* = 0.863, *p* ≤ 0.002 for protein and *r* = 0.728, *p* ≤ 0.05 for amino acid) and TFC (*r* = 0.817, *p* ≤ 0.05 for protein and *r* = 0.740, *p* ≤ 0.05) (Table 4). A similar correlation was predicted in Algerian honey samples [38]. Protein and amino acid contribute to the antioxidant potential of honey and these bioactive compounds strongly correlated with TEAC (*r* = 0.923, *p* ≤ 0.002 for protein and *r* = 0.899, *p* ≤ 0.01 for amino acid), DPPH (*r* = 0.772, *p* ≤ 0.01 for protein and *r* = 0.922, *p* ≤ 0.003 for amino acid), FRAP (*r* = 0.660, *p* ≤ 0.05 for protein and *r* = 0.694, *p* ≤ 0.05 for amino acid) and also with each other (*r* = 0.947, *p* ≤ 0.004) (Table 4). Our results are compatible with previously observed values of Malaysian [41], Algerian [38], Bangladeshi [44], and Indian honeys [46].

### 2.5. Cytotoxic Effects MH and STH on HCT-116, Lovo and HDFcells

**Figure 1:**
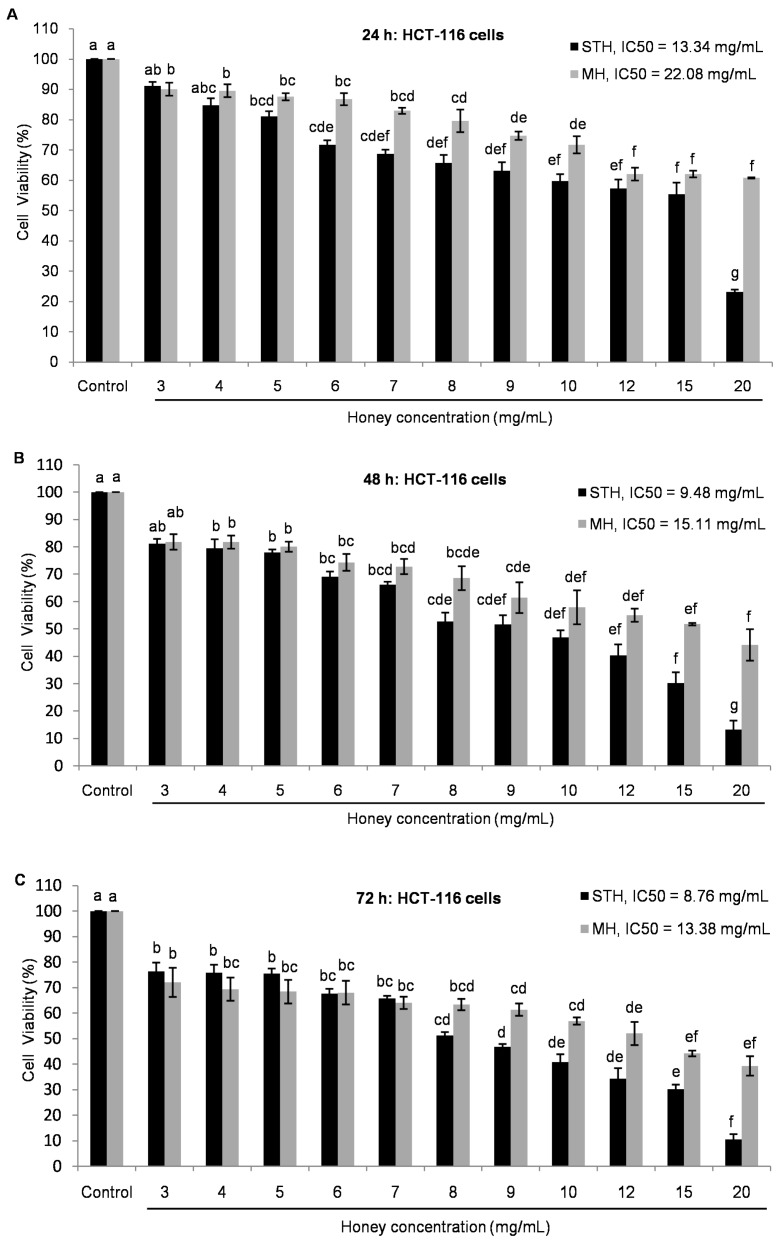
Inhibition of cell proliferation by strawberry tree honey (STH) and Manuka honey (MH) in HCT-116 cell lines (**A**–**C**). After 24 h of cell seeding, HCT-116 were treated with different concentrations of both honeys (0–20 mg/mL) for 24, 48 and 72 h. Cell viability was measured by using 3-(4,5-dimethylthiazol-2-yl)-2,5-diphenyltetrazolium bromide (MTT) assay and results were expressed as a percentage (%) of viable cells compared to control cells. Data are shown as the mean ± SD of three experiments. Different superscripts letter for each column indicated significant differences (*p* < 0.05).

As STH from Berchidda area has the highest amount of phytochemicals and antioxidant properties, we further decided to evaluate the cytotoxic effect of this STH in comparison with MH on HCT-116 and LoVo cells. To our knowledge, this study is the first that attempts to demonstrate the anticancer potential of STH and MH by evaluating cell proliferation and ROS production on human colon cancer cells (HCT-116 and LoVo cells). To investigate the cytotoxic effects of STH and MH on HCT-116 and LoVo cells, the 3-(4,5-dimethylthiazol-2-yl)-2,5-diphenyltetrazolium bromide (MTT) assay was performed. Cells were treated for 24, 48 or 72 h with various concentrations of STH and MH ranging from 3 to 20 mg/mL for HCT-116 cells and 5 to 60 mg/mL for LoVo cells. The range of concentrations used to treat the LoVo cells was higher than the range used for HCT-116, because of its metastatic nature. It was noted that, in LoVo cells at a lower concentration (3 and 4 mg/mL), there was no significant cytotoxic effect. In HCT-116 cells, the IC_50_ (concentrations required for 50% inhibition of cell growth) of STH and MH were 13.34 and 22.08 mg/mL at 24 h, 9.48 and 15.11 mg/mL at 48 h, and 8.76 and 13.38 mg/mL at 72 h, respectively (Figure 1).

In a previous study, the IC_50_ value of MH ranged between 20 and 25 mg/mL against colon cancer CT-29 cells at 24 h [29]. Likewise, the IC_50_ value was 15.11 mg/mL in MH for HCT-116 cells in the present study. Hakim et al. reported an IC_50_ value of Gelam honey from Malaysia that was 75 mg/mL on HCT-116 cells [34]. Furthermore, using the same honey, the IC_50_ values were observed as 39, 80 and 88 mg/mL, respectively, on other colon cancer cells by a different research group [31,32,33].

**Figure 2:**
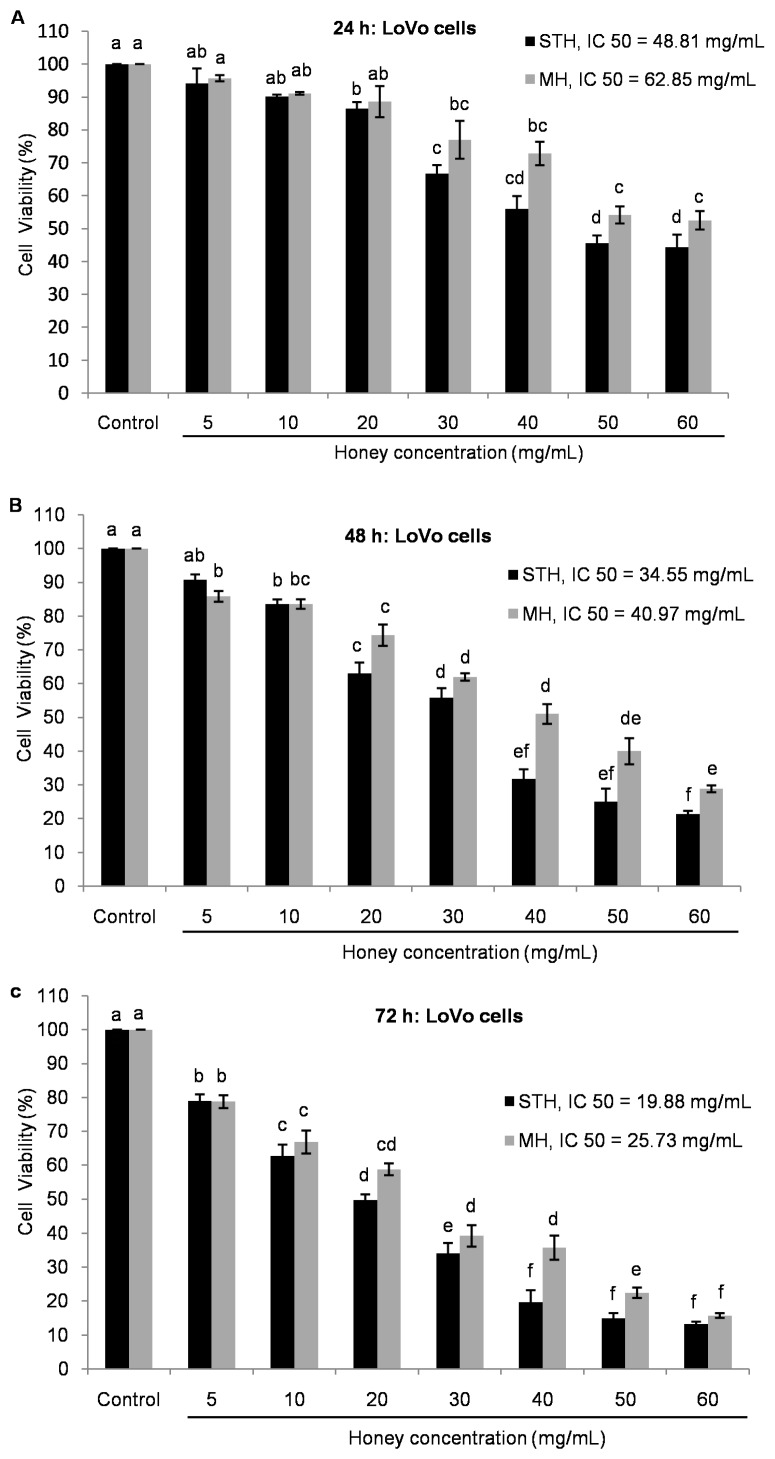
Inhibition of cell proliferation by STH and MH in LoVo cell lines (**A**–**C**). After 24 h of cell seeding, LoVo were treated with different concentrations of both honeys (0–60 mg/mL) for 24, 48 and 72 h. Cell viability was measured by using MTT assay and results were expressed as a percentage (%) of viable cells compared to control cells. Data are shown as the mean ± SD of three experiments. Different superscripts letter for each column indicated significant differences (*p* < 0.05).

In LoVo cells, the IC_50_ concentration of STH and MH were 48.81 and 62.85 mg/mL at 24 h, 34.55 and 40.97 mg/mL at 48 h and 19.88 and 25.73 mg/mL at 72 h, respectively (Figure 2).

As shown in Figure 1 and Figure 2, STH and MH decreased cell viability in a dose and time dependent manner. In both cell lines, the treatment with STH caused a greater decrease on cellular viability at lower concentrations than MH. To evaluate the anti-proliferative effect of honey on colon cancer cell line, it was necessary to use different concentrations, most likely due to variations in honey content, particularly in polyphenols and antioxidant activities [6,7,47].

**Figure 3:**
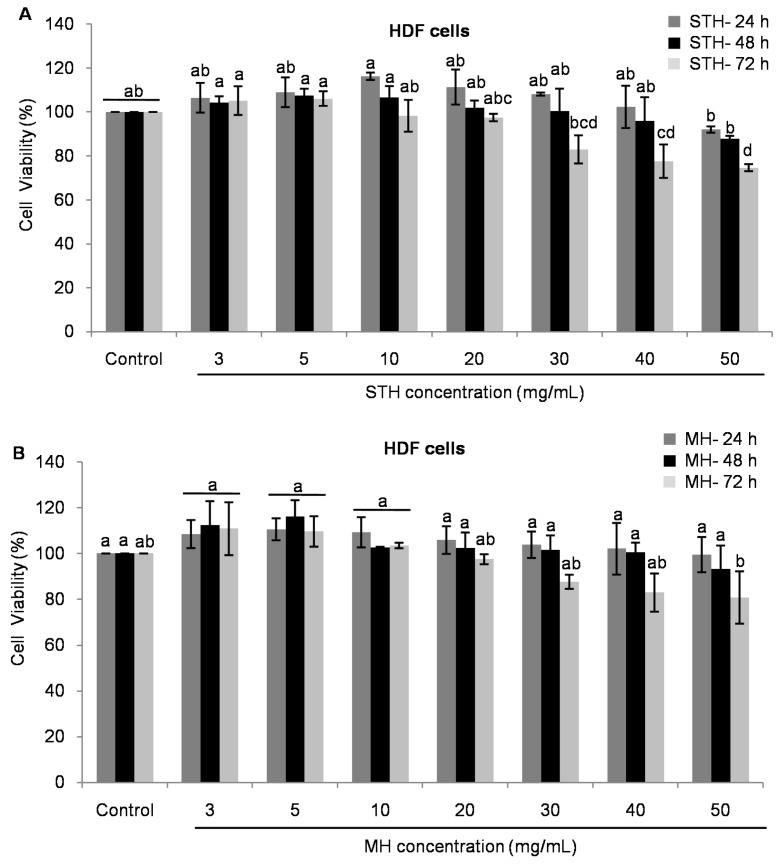
Effects of STH and MH on cell viability of HDF cells (**A**,**B**). After 24 h of cell seeding, HDF were treated with different concentrations of both honeys (0–50 mg/mL) for 24, 48 and 72 h. Cell viability was measured by using MTT assay and results were expressed as a percentage (%) of viable cells compared to control cells. Data are shown as the mean ± SD of three experiments. Different superscripts letter for each column indicated significant differences (*p* < 0.05).

In the case of normal HDF, STH and MH exhibited no toxic effects compared to control until 48 h in the concentrations from 3 to 40 mg/mL (Figure 3A,B). After 72 h, the cell viability was affected in the concentrations of 20 mg/mL for STH and 50 mg/mL for MH by inducing less toxic effects compared to control (Figure 3). In all cases, normal HDF were significantly (*p* < 0.05) less toxic to both honeys compared to colon cancer HCT-116 and LoVo cells.

**Figure 4:**
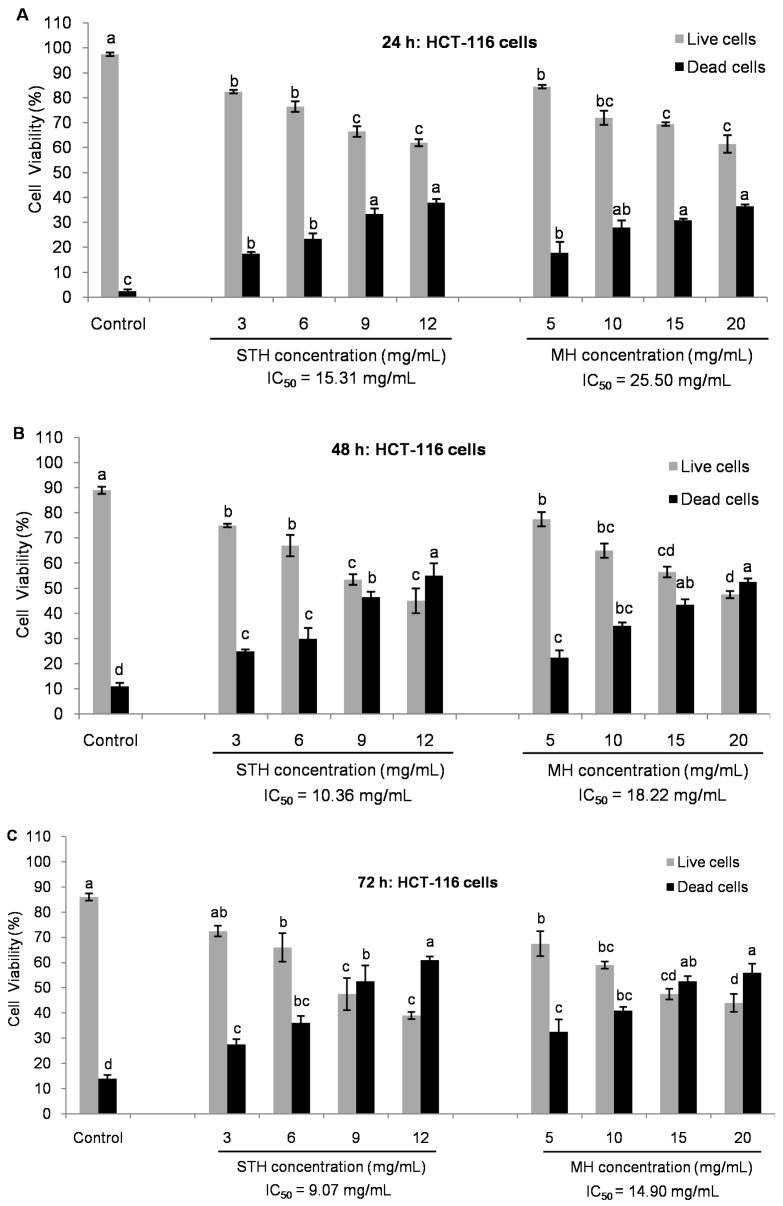
Comparison of viability in cell populations between STH and MH in HCT-116 cells by Tali™ Image-Based Cytometer (**A**–**C**). After 24 h of cell seeding, HCT-116 cells were treated with STH (0, 3, 6, 9 and 12 mg/mL) and MH (0, 5, 10, 15 and 20 mg/mL) for 24, 48 and 72 h at which time approximately 80% to 40% cells were alive. Cell viability was measured by using Tali™ Viability Kit assay and results were expressed as a percentage (%) of live and dead cells. Data are shown as the mean ± SD of three experiments. Different superscripts letter for each column indicated significant differences (*p* < 0.05).

**Figure 5:**
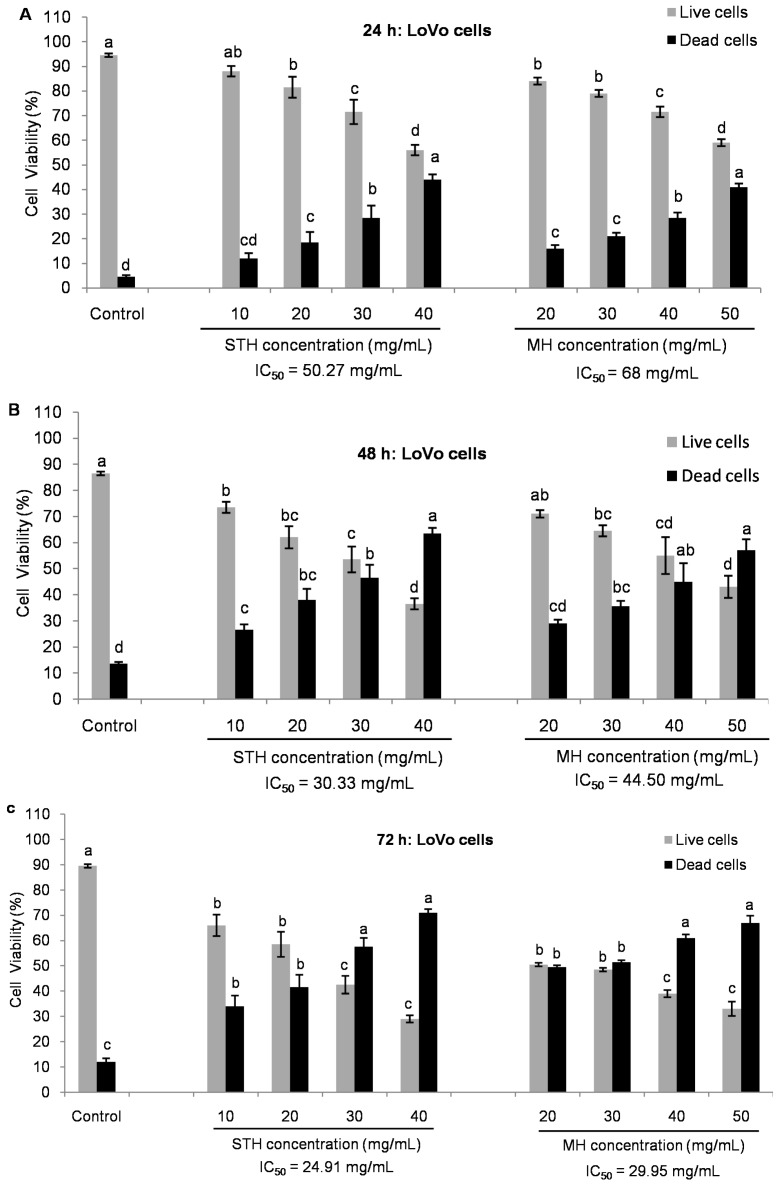
Comparison of viability in cell populations between STH and MH in LoVo cells by Tali™ Image-Based Cytometer (**A**–**C**). After 24 h of cell seeding, LoVo cells were treated with STH (0, 10, 20, 30 and 40 mg/mL) and MH (0, 20, 30, 40 and 50 mg/mL) for 24, 48 and 72 h at which approximately 80% to 30% cells were alive. Cell viability was measured by using Tali™ Viability Kit assay and results were expressed as a percentage (%) of live and dead cells. Data are shown as the mean ± SD of three experiments. Different superscripts letter for each column indicated significant differences (*p* < 0.05).

To confirm the number and proportion of viable and dead cells after honey treatment, the TALI^®^ viability assay was also carried out. Prior to this analysis those concentrations at which approximately 80% to 40% cells were viable by MTT assay were selected. We found that both honey treatments significantly decreased cell viability rate which was quite similar to the data obtained with the MTT assay, with minor differences (Figure 4 and Figure 5). At 24, 48 and 72 h, the IC_50_ values for HCT-116 cells were 15.31, 10.36 and 9.07 mg/mL for STH and 25.50, 18.22 and 14.90 mg/mL for MH (Figure 4), respectively.

In LoVo cells, the IC_50_ values were 50.27 mg/mL at 24 h, 30.33 mg/mL at 48 h and 24.91 mg/mL at 72 h for STH and for MH the values were 68 mg/mL at 24 h, 44.50 mg/mL at 48 h and 29.95 mg/mL at 72 h, correspondingly (Figure 5).

Based on the cell viability data, the IC_50_ concentration of STH was lower than MH, suggesting that STH from Berchidda area may be superior from the point of view of anticancer potential compared to MH.

### 2.6. Intracellular ROS Production by STH and MH on HCT-116 and LoVo Cells

**Figure 6:**
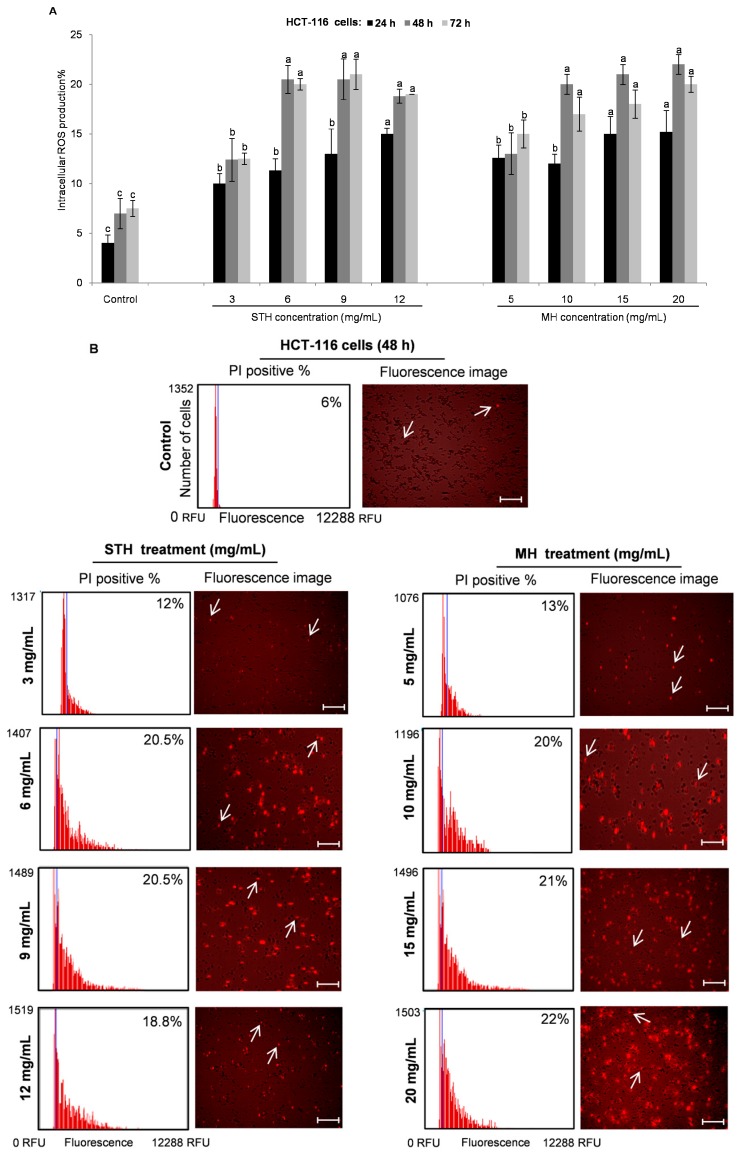
STH and MH induce ROS generation in HCT-116 cells. HCT-116 cells were treated with or without different concentrations of STH (0, 3, 6, 9 and 12 mg/mL) and MH (0, 5, 10, 15 and 20 mg/mL)for 24, 48 and 72 h. Intracellular ROS levels were calculated by using CellROX^®^ Orange assay kit and the Tali™ Image-based Cytometer (**A**). Image-Based cytometry was used to quantify ROS induction (% of propidium iodide (PI) positive) in HCT-116 cells following STH and MH treatment at 48 h (**B**). The blue line of the thumbnail histogram indicated the set threshold. Representative fluorescence image of HCT-116 cells shows the effect of STH and MH treatment at 48 h: non-fluorescent while in a reduced state and bright red fluorescence upon oxidation by ROS. Scale bar = 50 µm, arrows indicate single cell (cell size = 10 µm). Data are shown as the mean ± SD of three experiments. Columns associated with the same set of data with different symbolic letters are significantly different (*p* < 0.05) from controls.

**Figure 7:**
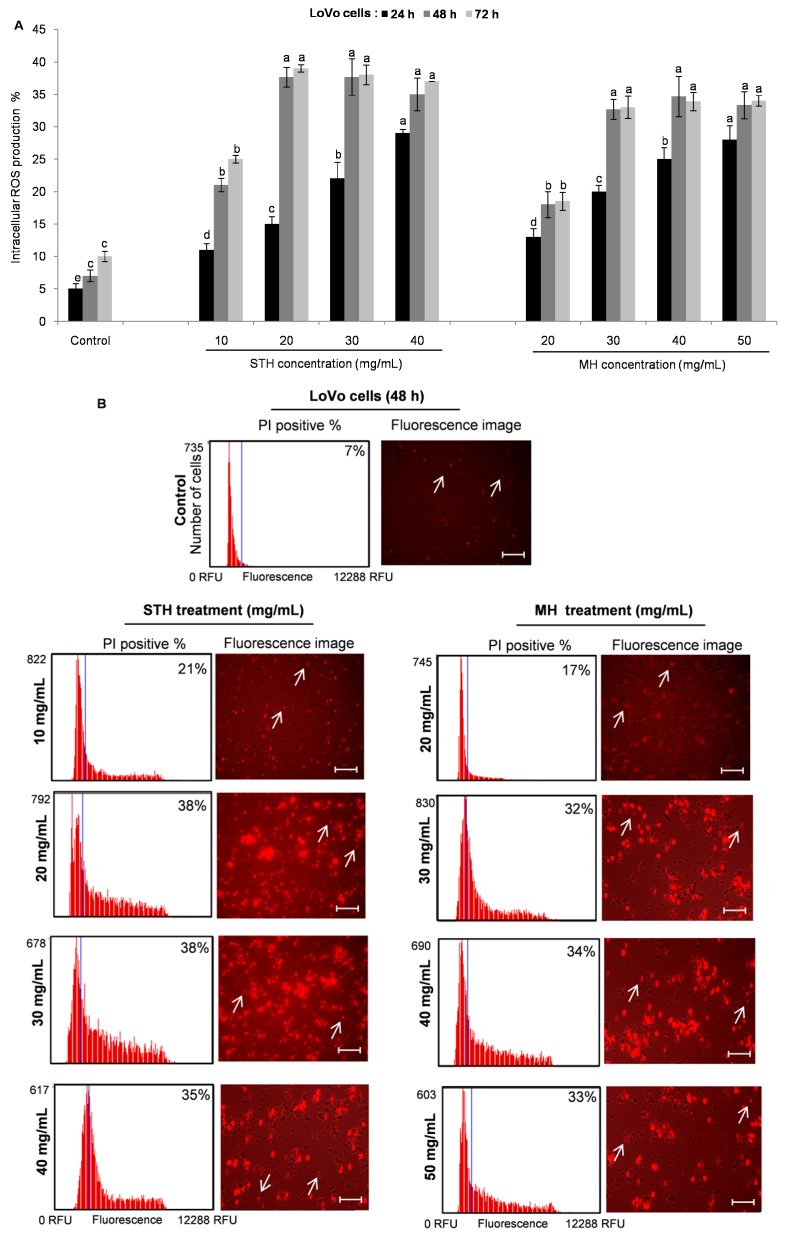
STH and MH induce ROS generation in LoVo cells. LoVo cells were treated with or without different concentrations of STH (0, 10, 20, 30 and 40 mg/mL) and MH (0, 20, 30, 40 and 50 mg/mL) for 24, 48 and 72 h. Intracellular ROS levels were calculated by using CellROX^®^ Orange assay kit and the Tali™ Image-based Cytometer (**A**). Image-Based cytometry was used to quantify ROS induction (% of PI positive) in LoVo cells following STH and MH (**B**) treatment at 48 h. The blue line of the thumbnail histogram indicated the set threshold. Representative fluorescence image of LoVo cells shows the effect of STH and MH treatment: non-fluorescent while in a reduced state and bright red fluorescence upon oxidation by ROS. Scale bar = 50 µm, arrows indicate single cell (cell size = 10 µm). Data are shown as the mean ± SD of three experiments. Columns associated with the same set of data with different symbolic letters are significantly different (*p* < 0.05) from controls.

ROS are largely described as molecules, ions or radicals including superoxide anion, organic and hydroxyl radicals, singlet oxygen and hydrogen peroxide, which are by-products of mitochondrial metabolism and redox signaling [48]. Depending on the concentration, ROS have a dual effect on cancer. Higher ROS levels have been found to play a role in tumor initiation and also perform a chemotherapeutic effect in suppressing cancer growth by promoting apoptosis and cell death [49]. In order to determine the intracellular ROS levels, HCT-116 and LoVo cells were treated with or without various concentrations of STH (3 to 12 mg/mL for HCT-116 cells and 10 to 40 mg/mL for LoVo cells) and MH (5 to 20 mg/mL for HCT-116 cells and 20 to 50 mg/mL for LoVo cells) for 24, 48 and 72 h, and analyzed using the CellROX^®^ Orange assay kit by Tali™ Image-based Cytometer. Both honeys significantly (*p* < 0.05) triggered intercellular ROS accumulation in HCT-116 and LoVo cells in a dose and time dependent manner (Figure 6 and Figure 7).

In HCT-116 cells, the highest percent of intracellular ROS production (22%)was at 48 h after treatment with MH (20 mg/mL) (Figure 6A), while ROS production was 20.5% at 72 h after treatment with STH (6 and 9 mg/mL) (Figure 6A). Fluorescence intensity showed a significant and dose-dependent increase in intracellular ROS levels in HCT-116 cells after being treated with STH and MH for 48 h (Figure 6B). In this case, ROS production was similar in both STH and MH treated HCT-116 cells. Moreover, in LoVo cells higher ROS percentage was 38% at 48 h treated with STH at 20 mg/mL, while MH induced 34% ROS at 40 mg/mL at the same time (Figure 7A). Similarly, fluorescence intensity was higher in LoVo cells treated with STH compared to MH (Figure 7B).

In the only paper to our knowledge, Indian commercial honey induced apoptotic cell death on colon cancer HCT-15 and HT-29 cells by increasing ROS generation [50]. Several studies have addressed the effect that dietary phytochemicals induced ROS generation for modulation of intracellular signaling cascades and triggered a series of programmed cell death pathways [51,52].The result suggests that both honey treatments induced ROS generation and STH induced more ROS generation in LoVo cells compared to MH.

## 3. Materials and Methods

### 3.1. Honey Samples

STH samples were collected from 5 different areas of Sardinia, Italy in 2014, namely Monti, Luras, Sadali, Olbia and Berchidda area. MH from New Zealand was used as a standard for comparison because it is a well-known honey worldwide and has been extensively studied. All samples were collected within their shelf life and were stored at 4 °C before analysis.

### 3.2. Chemicals and Reagents

Folin–Ciocalteu reagent, 6-hydroxy-2,5,7,8-tetramethylchromane-2-carboxylic acid (Trolox), bovine serum albumin (BSA), 2,2′-azinobis(3-ethylbenzothiazoline-6-sulfonic acid) (ABTS, diammonium salt) were purchased from FlukaChemie (Buchs, Switzerland). Sodium carbonate (Na_2_CO_3_), gallic acid, sodium nitrite (NaNO_2_), aluminum chloride (AlCl_3_), sodium hydroxide (NaOH), (+)-Catechin, methanol, Coomassie Brilliant Blue, phosphoric acid (H_3_PO_4_), sodium chloride (NaCl), ninhydrin, acetic acid, cadmium chloride hemi (pentahydrate), l-Leucine, 2,4,6-tripyridyl-*S*-triazine (TPTZ), ferric chloride (FeCl_3_), ammonium ferrous sulfate, potassium persulfate (K_2_SO_4_), and DPPH radicals were purchased from Sigma-Aldrich Chemie GmbH (Steinheim, Germany). Media and reagents for cell culture were purchased from ATCC and Carlo Erba Reagents (Milan, Italy). Tali™ Viability Kit-Dead Cell Green and CellROX^®^ Orange Reagents were purchased from Invitrogen TM, Life Technologies. Chemicals and solvents were of analytical grade.

### 3.3. Determination of TPC and TFC

Polyphenol and flavonoid contents of honey samples were determined according to procedures previously described by Alvarez-Suarez et al. [11] with minor modifications.

TPC was determined based on the Folin–Ciocalteu method. One gram of honey sample was dissolved in 10 mL distilled water and filtered through Minisart filter of 45 μm (PBI International). In 500 μL of filtered sample 2.5 mL of 0.2 N Folin–Ciocalteu reagents were added and kept 5 min at room temperature (RT). Then it was mixed with 0.7 M Na_2_CO_3_ and incubated in the dark at RT for 2 h. The absorbance was measured at 760 nm using a Beckman Du 640 spectrophotometer (Instruments Inc., Fullerton, CA, USA). Gallic acid was used as standard to calculate the calibration curve (50–300 mg/L). TPC was expressed as g of gallic acid equivalents (GAE) per kg of honey.

For determination of TFC, 250 μL of honey solution (50% *w*/*v* in methanol) was mixed with 1.25 mL distilled water and 75 μL of a 5% NaNO_2_ solution. After 6 min, 150 μL of 10% AlCl_3_·H_2_O solution was added, and after a wait of another 5 min 500 μL 1 M NaOH was added. Then the mixture was brought to 2.5 mL with the addition of distilled water and the absorbance was measured at 515 nm using a Beckman Du 640 spectrophotometer (Instruments Inc., Fullerton, CA, USA). (+)-Catechin was used as a standard to calculate the calibration curve (5–50 mg/L). TFC was expressed as mg of (+)-catechin equivalents (CAE) per kg of honey.

### 3.4. Determination of Total Protein and Free Amino acid Content

The protein content of honey was determined by Bradford’s method [53]. A 100-μL honey solution (50% *w*/*v* in methanol) was added to 5 mL of the Coomassie Brilliant Blue reagent mixture. The Coomassie Brilliant Blue formed a blue complex with the protein. After incubation (2 min), the absorbance was determined at 595 nm against the blank (the reactive solution without the sample) using a spectrophotometer (Beckman Du 640, Beckman, Brea, CA, USA). BSA was used as a standard for calculating the calibration curve (10–100 μg/0.1 mL) in 0.15 M NaCl. The protein content was expressed as g of bovine serum albumin (BSA) per 100 g of honey.

Free amino acid content was measured with the Cd-ninhydrin method as performed by Doi et al. [54]. The reaction solution consisted of 0.8 g of ninhydrin mixed in 80 mL of 99.5% ethanol and 10 mL of acetic acid, followed by adding a solution of 1.24 g of cadmium chloride hemi (pentahydrate) in 1 mL of distilled water. Honey sample (1.25 g) was diluted into 25 mL of distilled water. Next, 1 mL of honeysolution was added in 2 mL of the reaction solution and heated for 5 min at 84 °C, and then cooled in ice. The absorbance was determined at 507 nm against the blank (the same mixture without the sample) using a spectrophotometer (Beckman Du 640, Beckman). l-Leucine was employed for the calibration curve (1.2–42 mg/L), and free amino acid content was expressed as mg of l-Leucine equivalents (LE) per 100 g of honey.

### 3.5. Determination of TAC

TAC of honey sample was quantified by FRAP, TEAC and DPPH. The FRAP assay was performed according to a modified method as described by Benzie and Strain [55]. The principle of this method is based on the reduction of a ferric 2,4,6-tripyridyl-*S*-triazine complex (Fe^3+^-TPTZ) to its ferrous colored form (Fe^2+^-TPTZ) in the presence of antioxidants. One gram of honey sample was dissolved in 10 mL of distilled water and then 200 μL of diluted honey solution was mixed with 1.8 mL FRAP reagent. The fresh FRAP reagent contained 2.5 mL of a 10 mM TPTZ solution in 40 mM HCl, 2.5 mL of 20 mM FeCl_3_ and 25 mL of 0.3 M acetate buffer, pH 3.6 and kept in the dark at 37 °C. The reaction mixture was incubated at 37 °C for 10 min and the absorbance was measured at 593 nm using a Beckman Du 640 spectrophotometer (Instruments Inc., Fullerton, CA, USA). Trolox (15–200 mM) and ammonium ferrous sulfate (25–250 mM) was used as the standard to calculate the calibration curves. The results were expressed as mmoles of Trolox equivalents (TE) per 100 g of honey and mmoles of ammonium ferrous sulfate (Fe (II)) per 100 g of honey.

The TEAC assay was performed according to the method previously described by Re et al. [56]. This method is based on the ability of antioxidant compounds to quench the 2,2′-azino-bis(3-ethylbenzothiazoline-6-sulfonic acid) (ABTS) radical cation (ABTS^+^) and reduce the radical to the colorless neutral form. The solution of ABTS radical cation (ABTS^+^) was produced by reacting 7 mM ABTS aqueous stock solution with 2.45 mM K_2_SO_4_, and maintained in the dark at 25 °C for 12 h before use. Immediately before analysis, the working solution was obtained by diluting the stock solution with ethanol. One gram of honey sample was diluted in 1 mL distilled water, and then 10 μL of sample was added in 1 mL of ABTS^+^ working solution. The reaction mixture was incubated at RT for 90 s and the color inhibition of the ABTS^+^ radical was measured at 734 nm using a Beckman Du 640 spectrophotometer (Instruments Inc., Fullerton, CA, USA). The percentage of radical-scavenging activity (RSA) was calculated according the following equation: % RSA = (Abs control − Abs sample/Abs control) × 100, where Abs is the absorbance. Trolox was used for the calibration curve (50–500 μM), and the results were expressed as mmol of Trolox equivalents (TE) per 100 g of honey.

DPPH radical assay was performed to determine the free radical-scavenging activity of honey based on the modified method described by Ferreira et al. [37]. This method is based on the ability of DPPH to react with the phenolic compounds present in the honey sample. The DPPH radical is a persistent molecule, characterized by its violet color. One gram of honey is dissolved in 1 mL of distilled water then 300 μL of this solution is mixed with 2.7 mL of methanolic solution containing DPPH radicals (6 × 10^−5^ mol/L). The inhibition of the DPPH radical was calculated by measuring the absorption at 515 nm using a Beckman Du 640 spectrophotometer (Instruments Inc., Fullerton, CA, USA). The percentage of radical-scavenging activity (RSA) was calculated according the following equation: % RSA = (Abs control − Abs sample/Abs control) × 100, where Abs is the absorbance. Trolox was used for the calibration curve (50–500 μM), and the results were expressed as mmol of Trolox equivalents (TE) per 100 g of honey.

### 3.6. Cell Culture

Human colon carcinoma (HCT-116) and Dukes’ type C, grade IV, colon metastasis (LoVo) cell lines were purchased from the American Type Culture Collection (ATCC, Manassas, VA, USA), and normal human dermal fibroblast (HDF) were provided by GIBCO^®^ Invitrogen cells. HCT 116 was cultured in McCoy’s 5A media, LoVo was cultured in F12K medium, while HDF was cultured in DMEM media and the entire media were supplemented with 10% heat-inactivated fetal bovine serum as well as 100 IU/mL penicillin and 100 μg/mL streptomycin. All cells were maintained in a CO_2_ incubator at 37 °C under a humidified atmosphere (95% air, 5% CO_2_).

### 3.7. Determination of Cell Survival Rate by MTT Assay

Cells were seeded (5000 cells/well) in sterile 96-well plates in complete growth medium. They were incubated overnight to allow cell attachment. Following overnight incubation, the HCT-116 cells were treated with 3 to 20 mg/mL, LoVo cells were treated with 5 to 60 mg/mL andHDF cells were treated with 3 to 50 mg/mL concentrations of STH and MH, while control wells were treated only with medium. After 24, 48 and 72 h incubation, 30 μL of RPMI medium containing 2 mg/mL of MTT was added and cells were incubated for other 4 h. The generated formazan crystals were solubilized by adding 100 μL of DMSO, and quantified by a microplate reader (ThermoScientific Multiskan EX, Thermo Fisher Scientific, Waltham, MA, USA) at a wavelength of 590 nm. The percentage of viable was calculated as (absorbance of treated cells/absorbance of control cells) × 100.

### 3.8. Measuring Cell Viability by TALI^®^ Viability Assay

Cells were seeded (1.5 × 10^5^ cells/well) in 6 well plates in complete growth medium. The range of STH and MH concentrations was chosen according to the approximately 80% to 40% cells which were viable in the MTT viability assay. Following overnight incubation, the HCT-116 cells were treated with 2 mL of medium containing STH (0, 3, 6, 9 and 12 mg/mL) and MH (0, 5, 10, 15 and 20 mg/mL) for 24, 48 and 72 h. In the case of LoVo cells, the concentration of STH was 0, 10, 20, 30 and 40 mg/mL and the concentration of MH was 0, 20, 30, 40 and 50 mg/mL. Cell viability was determined by using the Tali™ Viability Kit—Dead Cell Green reagent following the manufacturer’s instructions and as previously reported by Rampele et al. [57].The proportions of viable and dead cells were analyzed by the Tali™ RFP + Viability assay on the Tali™ Image-Based Cytometer.

### 3.9. Determination of Intracellular ROS Generation

Intracellular ROS levels were determined by CellROX^®^ Oxidative Stress kit according to the manufacturer’s instructions. Cells (1.5 × 10^5^ cells/well) were incubated with STH (0, 3, 6, 9 and 12 mg/mL for HCT-116 cells and 0, 10, 20, 30 and 40 mg/mL for LoVo cells) and MH (0, 5, 10, 15 and 20 mg/mL for HCT-116 cells and 0, 20, 30, 40 and 50 mg/mL for LoVo cells) for 24, 48 and 72 h. After that, cells were trypsinaysed and centrifuged at 1500 rpm for 10 min. Then, 2 μL of CellROX^®^ Orange reagent was added and incubated 30 min at 37 °C. Medium was removed and cells were washed three times with PBS. Cells were analyzed with the Tali^®^ Image-Based cytometer, and the results were expressed as the percentage of cells with increased ROS levels compared with the control. The cell-permeable reagents are fluorescent while in an oxidation state and non-fluorescent while in a reduced state.

### 3.10. Statistical Analysis

The results are expressed as the mean values with standard deviations (SD) of three experiments and the statistical analysis was performed using STATISTICA software (Statsoft Inc., Tulsa, OK, USA). The significant differences represented by letters were obtained by a one-way analysis of variance (ANOVA) followed by Tukey’s honestly significant difference (HSD) post hoc test (*p* < 0.05). Correlations were determined on a honey mean basis, according to Pearson’s correlation coefficient (*r*). Differences at *p* ≤ 0.05 were considered to be statistically significant.

## 4. Conclusions

The present study demonstrated that STH and MH can induce cell death and increase intracellular ROS generation in colon cancer cells, and that bioactive compounds of honey depend on its floral sources, geographical origins, seasonal and environmental factors which have a significant impact on the antiproliferative and antioxidant potential. In our study, in fact, STH honey from Berchidda area induced more cytotoxic effects compared to MH, possibly due to its significant amount of phytochemical and antioxidant activity. Molecular studies elucidating the pathways for the chemo-preventive activity of this honey are underway in our laboratory. These persuasive results increase our knowledge of honey and could be useful for the development of a therapeutic candidate for targeting colon cancer.

## Figures and Tables

**Table 1:** Total polyphenol and flavonoid content of Strawberry tree and Manuka honey.

Type of Honey	Total Polyphenols	Total Flavonoids
	(g GAE/Kg)	(mg CAE/kg)
Strawberry Tree Honey		
Monti	0.86 ± 0.01 ^b^	92.68 ± 14.17 ^a,b^
Luras	0.77 ± 0.02 ^b,c^	69.96 ± 3.62 ^b,c^
Sadali	0.76 ± 0.02 ^b,c^	65.74 ± 2.50 ^c^
Olbia	0.69 ± 0.01 ^c^	66.18 ± 0.61 ^c^
Berchidda	1.00 ± 0.02 ^a^	108.20 ± 2.69 ^a^
Manuka Honey	0.89 ± 0.01 ^a,b^	71.90 ± 0.03 ^b,c^

GAE: Gallic acid equivalent; CAE: (+)-Catechin equivalents. Data are presented as mean ± standard deviation (SD) of three independent experiments. Different superscripts letters for each column indicated significant differences (*p* < 0.05).

**Table 2:** Total protein and free amino acid content of Strawberry tree and Manuka honey.

Type of Honey	Total Protein	Total Free Amino Acids
	(g BSA/100 g)	(mg LE/100 g)
Strawberry Tree Honey		
Monti	0.04 ± 0.00 ^b,c^	14.56 ± 0.93 ^b^
Luras	0.03 ± 0.00 ^c^	12.86 ± 0.05 ^b^
Sadali	0.03 ± 0.00 ^c^	10.28 ± 0.86 ^b^
Olbia	0.04 ± 0.01 ^b,c^	13.18 ± 1.35 ^b^
Berchidda	0.07 ± 0.00 ^a^	51.67 ± 9.64 ^a^
Manuka Honey	0.05 ± 0.00 ^a,b^	14.34 ±0.13 ^b^

BSA: Bovine serum albumin; LE: Leucine equivalents. Data are presented as mean ± SD of three independent experiments. Different superscripts letter for each column indicated significant differences (*p* < 0.05).

**Table 3:** Total antioxidant capacity of Strawberry tree and Manuka honey.

Type of Honey	FRAP Values		TEAC Values	DPPH Values
	mmol TE/100 g	mmol Fe(II)/100 g	mmol TE/100 g	mmol TE/100 g
Strawberry Tree Honey				
Monti	0.39 ± 0.00 ^b^	0.81 ± 0.00 ^b^	0.10 ± 0.00 ^c^	0.09 ± 0.00 ^b^
Luras	0.30 ± 0.00 ^c^	0.68 ± 0.00 ^c^	0.11 ± 0.00 ^c^	0.09 ± 0.00 ^b^
Sadali	0.24 ± 0.00 ^d^	0.63 ± 0.00 ^d^	0.11 ± 0.00 ^c^	0.09 ± 0.00 ^b^
Olbia	0.21 ± 0.00 ^e^	0.51 ± 0.00 ^e^	0.11 ± 0.00 ^c^	0.09 ± 0.00 ^b^
Berchidda	0.54 ± 0.00 ^a^	0.92 ± 0.02 ^a^	0.39 ± 0.01 ^a^	0.20 ± 0.01 ^a^
Manuka Honey	0.14 ± 0.00 ^f^	0.29 ± 0.00 ^f^	0.22 ± 0.00 ^b^	0.06 ± 0.00 ^c^

FRAP: ferric reducing antioxidant power assay; TEAC: Trolox equivalent antioxidant capacity assay; DPPH: Diphenyl-1-picrylhydrazyl assay; TE: Trolox equivalents; Fe(II): Ferrous ammonium sulfate. Data are presented as mean ± SD of three independent experiments. Different superscripts letter for each column indicated significant differences (*p* < 0.05).

**Table 4:** Correlation matrix (Pearson’s correlation coefficients) showing the interrelation between quantitative determinations in the Strawberry tree and Manuka honeys ^a^.

Variable	TPC	TFC	FRAP	TEAC	DPPH	Protein
TFC	0.856 *					
FRAP	0.663 *	0.878 **				
TEAC	0.870 **	0.678 *	0.586 ^ns^			
DPPH	0.678 *	0.796 **	0.807 **	0.704 **		
Protein	0.863 **	0.817 *	0.660 *	0.923 **	0.772 **	
Free AA	0.728 *	0.740 *	0.694 *	0.899 *	0.922 **	0.947 **

TPC: Total phenolic content; TFC: Total flavonoid content; FRAP: ferric reducing antioxidant power assay; TEAC: Trolox equivalent antioxidant capacity assay; DPPH: Diphenyl-1-picrylhydrazyl assay; Free AA: Free amino acid. ^a^ 95% confidence interval, * Significant at *p* ≤ 0.05, ** Significant at *p* ≤ 0.01, ^ns^ non significant.
